# Loading Dynamics of a Sliding DNA Clamp**

**DOI:** 10.1002/anie.201403063

**Published:** 2014-05-22

**Authors:** Won-Ki Cho, Slobodan Jergic, Daehyung Kim, Nicholas E Dixon, Jong-Bong Lee

**Affiliations:** Department of Physics, School of Interdisciplinary Bioscience and Bioengineering, Pohang University of Science and Technology (POSTECH)Pohang (Korea); Centre for Medical and Molecular Bioscience, School of Chemistry, University of WollongongWollongong, N.S.W. 2522 (Australia)

**Keywords:** DNA clamps, DNA replication, single-molecule FRET, single-molecule polarization, ternary complexes

## Abstract

Sliding DNA clamps are loaded at a ss/dsDNA junction by a clamp loader that depends on ATP binding for clamp opening. Sequential ATP hydrolysis results in closure of the clamp so that it completely encircles and diffuses on dsDNA. We followed events during loading of an *E. coli* β clamp in real time by using single-molecule FRET (smFRET). Three successive FRET states were retained for 0.3 s, 0.7 s, and 9 min: Hydrolysis of the first ATP molecule by the γ clamp loader resulted in closure of the clamp in 0.3 s, and after 0.7 s in the closed conformation, the clamp was released to diffuse on the dsDNA for at least 9 min. An additional single-molecule polarization study revealed that the interfacial domain of the clamp rotated in plane by approximately 8° during clamp closure. The single-molecule polarization and FRET studies thus revealed the real-time dynamics of the ATP-hydrolysis-dependent 3D conformational change of the β clamp during loading at a ss/dsDNA junction.

The DNA clamps found in bacteria (β subunit of DNA polymerase III), archaea (archaeal proliferating cell nuclear antigen, PCNA), some phages (T4 gp45), and eukaryotes (PCNA) dramatically increase the processivity of DNA synthesis through direct interaction with polymerases.^1^ The clamps are ring-shaped oligomeric proteins with six similar domains that in their closed conformation completely encircle and diffuse on double-stranded (ds) DNA. For their loading, clamps need to be opened and then closed with defined polarity around a primer–template junction in an adenosine triphosphate (ATP)-dependent manner by a specialized clamp loader (γ/τ complex in bacteria, replication factor C in archaea and eukaryotes), which opens the clamp to place it onto DNA.^2^ The clamps thus have two distinct faces: an N-terminal face towards the exiting dsDNA and a C-terminal face that contains a hydrophobic pocket at which a variety of clamp-binding proteins, including the clamp loader and polymerase, interact (Figure 1 a).1e,f, 3

**Figure 1:**
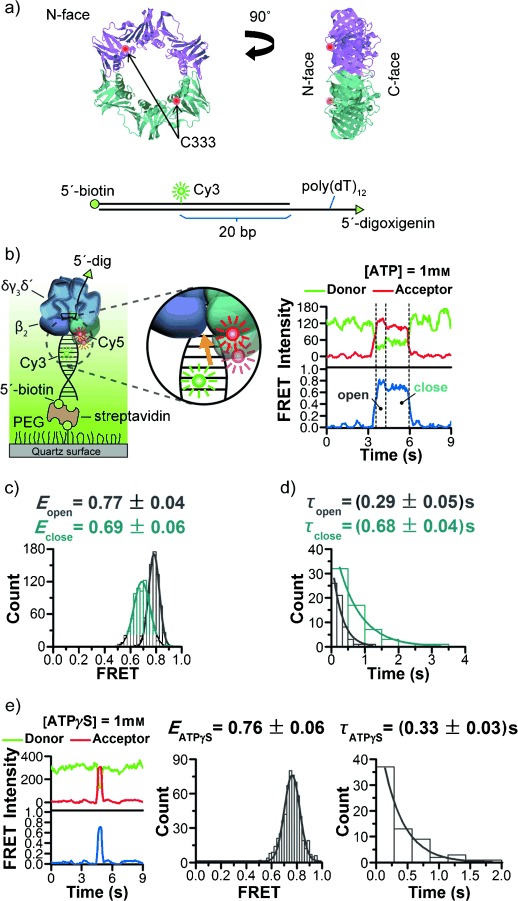
Loading of the *E. coli* β_2_ clamp onto DNA by the γ clamp loader. a) A fluorescent acceptor (Cy5) was attached at C333 on the N-terminal face of one protomer of β_2_ (PDB ID: 2POL). A DNA primer–template has a recessed 3′ primer terminus where the β_2_–γ complex is organized. A donor (Cy3) was attached to a base located 20 bp before the primer terminus. b) Schematic representation of the ternary complex and the conformation change of the clamp (PEG=poly(ethylene glycol)), and representative trajectories of the fluorescence intensity and FRET efficiency in the presence of 5 nm β_2_, 5 nm δγ_3_δ′, and 1 mm ATP. c) Corresponding distributions of FRET efficiency (mean±s.d.) and d) dwell time (mean±s.d.) of two distinct FRET states obtained from *N*=61 molecules. e) Representative trajectories of fluorescence intensity and FRET efficiency as well as distributions of FRET efficiency and dwell time (*N*=68) in the presence of 1 mm ATPγS.

The C-terminal domains of the five structurally related clamp-loader subunits form a cylindrical collar, and in a process dependent on ATP binding, their N-terminal domains engage the clamp.1f, 4 Docking of the ATP-bound clamp loader onto the C-face of the clamp opens one of the clamp interfaces by approximately 2.3 nm to provide enough space for DNA to enter.^5^ This process stimulates the hydrolysis of up to three ATP molecules by the clamp loader to enable closing of the clamp to encircle the DNA and subsequent dissociation of the loader.2a, 6 The circular clamp is able then to randomly translocate on the DNA by thermal fluctuation, thus effectively enabling its association with clamp-interacting proteins.

Biochemical and structural studies of a clamp–loader complex suggested that the hydrolysis of the first ATP molecule provokes closure of the clamp; the loader remains tethered to the closed clamp, and hydrolysis of the remaining ATP molecules leads to its complete detachment.2a, 5, 6c, 7 The kinetics of an ATP-hydrolysis-dependent conformational change shows discrete steps between clamp closing and its release on DNA.7a Although several single-molecule analyses of DNA sliding clamps have been performed,^8^ the ATP-dependent conformational change of the clamps when they are loaded onto DNA has not been observed directly. Understanding of the dynamic features of interactions in the ternary clamp–DNA–loader complex in the presence of ATP is critical for understanding of the clamp-loading mechanism.

In this study, we visualized the sequential steps during loading of the dimeric *Escherichia coli* β_2_ clamp onto a DNA primer template by the γ clamp loader (δγ_3_δ′) by using single-molecule Förster resonance energy transfer (smFRET). This investigation revealed the dynamics of the ATP-hydrolysis-dependent conformation change in β_2_ during its loading. By exploiting single-molecule polarization, we also imaged the rotational dynamics of a β_2_ interfacial domain during its transition from an open to a closed ring.

According to the crystal structure of the T4 clamp–clamp-loader complex, clamp closure results in the movement of interfacial domains 2 and 5 of the gp45 trimer by 2.3 nm along the DNA helical axis.^5^ A similar distance change would enable us to observe the closure of β_2_ during loading by smFRET. An acceptor fluorophore (Cy5) was conjugated to a Cys residue (C333) exposed on the N-terminal face of β_2_ (Figure 1 a). There are three other cysteine residues in each β protomer, but maleimide derivatives show a strong preference for C333;^9^ only 18 % of labeled β dimers contained more than two maleimide dyes (see Figure S1 in the Supporting Information). A single donor (Cy3) was attached to a partial-duplex DNA molecule (36 bp) with a 5′-dT_12_ overhang at the 20th base from the 3′ end of the primer (Figure 1 a; see Table S1 in the Supporting Information). Because the footprint of the complex of β_2_ with δγ_3_δ′ on DNA (ternary complex) is 16 bp,^10^ the distance between Cy3 and Cy5 in the Cy3–Cy5 pair is predicted to be 4 nm when β_2_–δγ_3_δ′ is bound at the primer terminus; the corresponding FRET efficiency (*E*) is expected to be 0.8. The DNA molecules were immobilized on a biotin–PEG-coated surface through a streptavidin linker (Figure 1 b, left). We determined the number of Cy5 residues on β_2_ by a photobleaching test with a strong 633 nm laser (700 mW cm^−2^) for the last 20 s of the imaging process, and only FRET signals from a single donor–acceptor pair were accepted for analysis.

A representative time trajectory with β_2_, δγ_3_δ′, and ATP under continuous laser excitation of Cy3 at 140 mW cm^−2^ showed two distinguishable FRET states until the fluorescence signals abruptly disappeared at 6 s (Figure 1 b, right). The FRET efficiency of the first state (*E*_open_) was 0.77±0.04 (Figure 1 c), as predicted for β_2_–δγ_3_δ′ bound at the primer terminus. After (0.29±0.05) s (*τ*_open_; Figure 1 d), the FRET efficiency decreased to a lower value (*E*_closed_=0.69±0.06; Figure 1 c), and this state was retained for (0.68±0.04) s (*τ*_closed_; Figure 1 d). Observation of the clamp on DNA was strictly dependent on the presence of ATP, and the FRET transition was observed in approximately 50 % of trajectories. This percentage probably reflects the two possible positions of Cy5 in the β dimer, only one of which will be near the open dimer interface. When a nonhydrolyzable ATP analogue (5′-[γ-thio]triphosphate, ATPγS) was used instead, there was no FRET transition; only the first, higher FRET state was seen (Figure 1 e). This result indicates that the FRET transition depends on ATP hydrolysis by the γ clamp loader. The dwell time with ATPγS (*τ*_ATPγS_) was nearly identical to the *τ*_open_ value with ATP (Figure 1 d and Figure 1 e, right), which might indicate a fine balance between dissociation into solution and ATP-hydrolysis-dependent clamp closure in the ternary complex.

To further study the loading dynamics, we end-blocked the 36 bp+dT_12_ primer template with an anti-digoxigenin antibody, which forces β_2_ to be retained and move on the DNA (Figure 2 a, left). This structure resulted in a third FRET state following the two observed for the unblocked DNA (Figure 2 a, right). The FRET values and dwell times in the first two states were nearly identical to those with unblocked DNA (Figures 1 and 2 b; see also Figure S2 a). The new, third state had a lower FRET efficiency (*E*_diffusion_=0.51±0.07; Figure 2 c) and usually persisted until the end of image acquisition (100 s). Therefore, its long dwell time was measured in a time-lapse experiment (*τ*_diffusion_=(9.1±0.6) min; see Figure S2 b) corresponding to a minimum estimate of the dwell time of β_2_ on dsDNA considering Cy3 photobleaching. Interestingly, the FRET signal of this state decreased to *E*_diffusion_=0.37±0.06 on a longer DNA molecule (75 bp+dT_10_) and disappeared quickly without the end block (see Figure S2 c). These results suggest that the β clamp might move rapidly along the DNA after the dissociation of δγ_3_δ′ from the ternary complex.^11^

**Figure 2:**
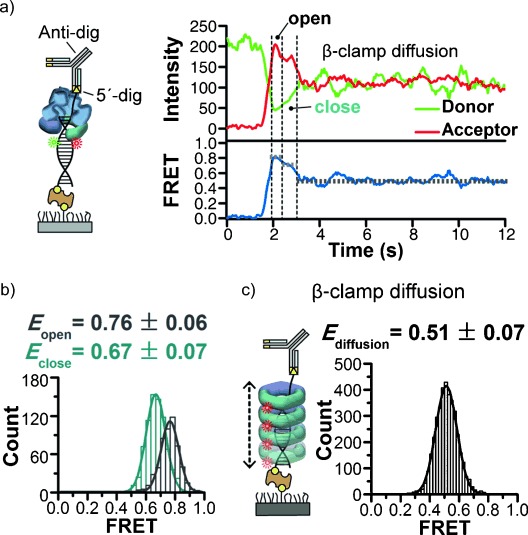
Dynamics of the β_2_ clamp on end-blocked DNA. a) One end of the DNA template was blocked with an anti-digoxigenin antibody. Representative trajectories of the fluorescence intensity and FRET efficiency show a third FRET state. b) Distributions of the FRET efficiency of the two states prior to the third (*N*=68). c) FRET efficiency of the third state (*N*=113).

The rapid diffusion of β_2_ on dsDNA was confirmed by the visualization of individual clamps moving on phage λ DNA (48.5 kb) with a diffusion coefficient *D*=(0.134±0.009) μm^2^ s^−1^ (in 0.1 m NaCl; see Figure S3), thus indicating that β_2_ would travel the 26 bp effective diffusion length of the 36 bp dsDNA molecules in 0.3 ms (faster than our time resolution of 30 ms). Therefore, the value of *E*_diffusion_ of 0.51 resulted from the time-averaged emission of donor–acceptor pairs while β_2_ diffused along the entire DNA length. With no intermediate FRET state observed in the presence of ATPγS, the FRET experiment provides dynamic insight into the open-to-closed conformational change of the clamp during its loading on DNA.

Domains 2 and 5 of the T4 clamp (corresponding to the location of Cy5 in β_2_) in its open conformation are separated by 0.9 nm in plane and 2.3 nm out of plane.^5^ Thus, when the clamp closes, 3D movement may result in rotation of the interfacial domains. Single-molecule polarization microscopy^12^ was used to resolve the rotational dynamics of the interfacial domains of β_2_ during its closing transition. The steady-state polarization (*P*) of a fluorophore is defined by the ratio of *I_H_*−*I_V_* to *I_H_*+*I_V_*, in which *I_H_* and *I_V_* are the emission intensities horizontal and vertical to the microscope stage. Cy3 conjugated to C333 of β_2_ was used as a dipole probe excited by circularly polarized incident light (Figure 3 a, left; see the Supporting Information). Our previous studies showed that the linkages maleimide–Cys for Cy3 protein labeling and biotin–streptavidin for DNA immobilization together reduced the rotational freedom of Cy3 on the surface-immobilized protein and DNA;12a, 13 this phenomenon enabled us to obtain polarized emissions of Cy3 on DNA-bound β_2_.

**Figure 3:**
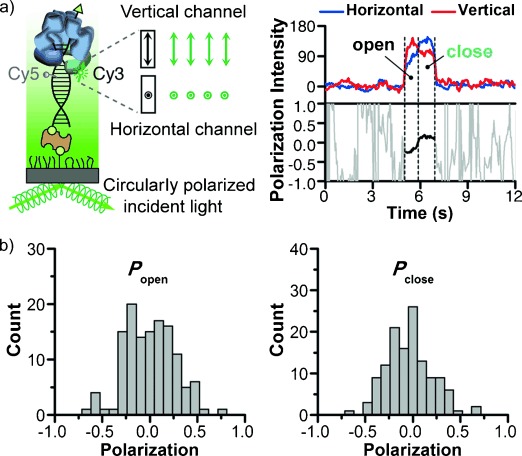
Single-molecule polarization. a) Schematic representation of single-molecule polarization and a representative trajectory of the fluorescence intensity and polarization (*P*). b) *P* values of two polarized states (*N*=128).

To identify DNA molecules bound to Cy3–β_2_, Cy5 attached to DNA was first imaged and completely photobleached to avoid any FRET. A representative polarization–time trajectory of a Cy3–β_2_ clamp colocalized with a 36 bp+dT_12_ DNA molecule displayed a transition between two polarized states at 6 s (Figure 3 a, right). The dwell times of each state were very close to those of clamp opening and closing as measured by FRET (Figure 1 d; see also Figure S4 in the Supporting Information). The broad distribution of the polarization of both states from individual traces indicates that β_2_ in both states of the ternary complex is rotationally constrained on the DNA, but that the ternary complex is randomly oriented on the surface (Figure 3 b). In contrast, after the dissociation of δγ_3_δ′, the emission of Cy3–β_2_ was depolarized (*P*_diffusion_=0.02±0.07; see Figure S5), thus indicating free rotation of the clamp around the DNA.12a The transition occurred in 49 % of the events with singly labeled β_2_, thus indicating that the polarization transition is only observed when the Cy3 label is on the interfacial domain of the β_2_ dimer that is opened by the clamp loader (see Figure S6). We conclude that the interfacial domain including the Cy3–C333 residue undergoes a rotational in-plane movement during closure of the clamp in the ternary complex.

To further analyze rotational dynamics, we determined the angle (*θ*) between the in-plane transition dipole and a transmission axis parallel to the horizontal channel (Figure 4 a). From the Malus law, *I_H_*=*I*_0_ cos^2^*θ* and *I_V_*=*I*_0_ sin^2^*θ*, in which *I*_0_ is the total emission intensity of the in-plane projection (Figure 4 a). The resulting polarization, *P*=1−2 sin^2^*θ*, enables determination of the angle difference (Δ*θ*) between the two distinct polarization states from an individual polarization trajectory (Figure 4 a). The distribution of Δ*θ* showed two symmetrical peaks with Δ*θ*=(−8.3±3.1)° and (+8.0±2.9)° (mean±standard deviation, s.d.; Figure 4 b). We interpret the two identical angle differences with different signs as arising because the transition dipole of Cy3–β_2_ can be located in any quadrant in the horizontal–vertical plane owing to the arbitrary orientation of the DNA molecule on the surface. For example, if the Cy3 domain of β_2_ moves clockwise during the conformational change, Δ*θ* of the in-plane dipole of Cy3 located in the first and third quadrants is positive, but Δ*θ* in the other two quadrants is negative. Therefore, the result suggests that the interfacial domain (domain 3) of β_2_ that includes Cy3–C333 rotates approximately 8° in plane when the clamp closes.

**Figure 4:**
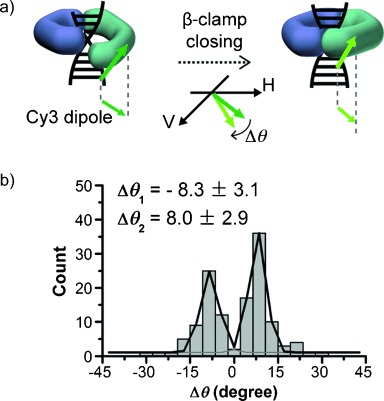
Difference in the rotational angle between two polarized states. a) Cy3 transition dipole in the horizontal and vertical planes. b) Symmetrical double-peaked Gaussian distribution of the angle difference.

In this study, we visualized the real-time conformational change of individual β_2_ clamps as they were loaded onto DNA by the clamp loader through smFRET detection of the distance change between DNA and the bound clamp, and also the rotation of the interfacial domain of β_2_ around DNA by single-molecule polarization. We have shown that smFRET combined with single-molecule polarization is a valuable tool for probing the dynamics of biomolecules on the basis of distance and orientational information. On the basis of the FRET decrease and the polarization change during the loading of β_2_, the clamp domain bound to the δ subunit of the δγ_3_δ′ loader moves in the out-of-plane direction and rotates outward by approximately 8° in plane as it opens (Figure 4 a). Jeruzalmi et al.^4^ showed that binding to the δ subunit of the γ clamp loader changed the curvature of domain 3 of a monomeric β mutant by 5° around the center of domain 2. Therefore, this rotation may correspond to an in-plane movement of approximately 0.7 nm, considering the length of domain 3 and half of domain 2 (4.7 nm).

Although the three γ subunits of the loader bind and hydrolyze ATP,^4^ only two FRET states were observed during clamp loading, and the second was twice as long as the first. It is likely that the hydrolysis of one^5^ or more7a ATP molecules results in closure of the clamp, and the remaining ATP molecule(s) are hydrolyzed in its closed conformation to enable dissociation of the loader from the ternary complex,^5^ thus liberating the closed clamp to diffuse on the primer–template DNA. We measured the total loading time of the clamp to be about 1 s, which is on the same time scale as Okazaki fragment synthesis on the lagging strand during DNA replication.
