# Supplementary material for: Loading Dynamics of a Sliding DNA Clamp
Source: Angew Chem Int Ed Engl. 2014 May 22;53(26):6768–71. doi: 10.1002/anie.201403063 (PMC4320747; doi:10.1002/anie.201403063)
Supplement: Supplementary file 1 [file anie0053-6768-sd1.pdf]

Angewandte Chemie
Eine Zeitschrift der Gesellschaft Deutscher Chemiker

Supporting Information
© Wiley-VCH 2014
69451 Weinheim, Germany

Loading Dynamics of a Sliding DNA Clamp**

*Won-Ki Cho, Slobodan Jergic, Daehyung Kim, Nicholas E. Dixon, and Jong-Bong Lee**

anie_201403063_sm_miscellaneous_information.pdf

Supporting Information

EXPERIMENTAL PROCEDURES

Proteins and Labeling

<u>Proteins</u>: The wild-type *E. coli* β_2 clamp[S1] and minimal clamp loader ($\delta\gamma_3\delta'$) complex[S2] were produced as described. Labeling reactions with dye-maleimides were modified from protocols described by Griep and McHenry.[S3]

<u>Buffers</u>: A: 50 mM Na-HEPES, pH 7.1, 0.5 mM EDTA; B: 50 mM Tris-HCl, pH 7.6, 3 mM dithiothreitol (DTT), 1 mM EDTA, 100 mM NaCl, 10% (v/v) glycerol; C: 40 mM Na-HEPES, pH 7.1, 2 mM tris(2-carboxyethyl)phosphine (TCEP), 0.5 mM EDTA, 20 mM NaCl; D: 40 mM Na-HEPES pH 7.1, 10 mM DTT, 0.5 mM EDTA, 20 mM NaCl, 20% (v/v) glycerol; E: 40 mM Tris-HCl pH 7.6, 3 mM DTT, 1 mM EDTA, 50 mM NaCl, 30% (v/v) glycerol.

<u>*Cy5-labeled β_2 (Cy5-β_2)*</u>: For buffer exchange, 1 mL of β_2 at 40 μM was passed at 1 mL/min through a column (1.5 x 12 cm) of Sephadex G-50 (GE Healthcare) equilibrated with buffer A + 200 mM NaCl. To the pooled void volume fractions (1.6 mL, 16 μM β_2) was immediately added 1 mg of Cy5-maleimide (GE Healthcare) in 80 μL neat *N*,*N*-dimethylformamide (DMF). The labeling reaction was allowed to proceed at room temperature for 2 h and then at 4˚C overnight. DTT was added to 5 mM, and excess Cy5 was removed from β_2 by gel filtration in buffer B, as above. A blue precipitate (characteristic of Cy5) observed during gel filtration was coincident with some protein loss. Fractions in the void volume containing Cy5-β_2 were pooled, frozen in liquid N_2 and stored at –80˚C. The presence of singly and doubly Cy5-labeled β_2 protomers, as well as unlabeled β_2 was confirmed by ESI-MS in 0.1% formic acid.

<u>*Cy3-labeled β_2 (Cy3-β_2)*</u>: 3 mL of β_2 (40 μM) was dialyzed overnight in buffer C. To ensure the reduced state of cysteines, TCEP and DTT concentrations were adjusted to 5 mM and 2 mM, respectively, and the solution left at room temperature for 3 h. β_2 was concentrated to 240 μM (0.5 mL) using an Amicon Ultra 0.5 mL centrifugal filter (Millipore), and then gel filtered as above in a column equilibrated with buffer A + 20 mM NaCl. To the pooled void volume fractions (2 mL, 35 μM β_2) was immediately added 1 mg of Cy3-maleimide (GE Healthcare) in 80 μL neat DMF. After 3h at room temperature and 4˚C overnight, DTT was added to 30 mM. After 1 h at 4˚C, excess Cy3 dye was removed by gel filtration in buffer D as above, except the sample volume was reduced to 0.6 mL prior to loading. This time, no precipitation was observed on the column. Fractions in the void volume containing Cy3-β_2 were pooled, dialyzed against one change of 1 L of buffer E, and stored in aliquots at –80˚C. The presence of singly Cy3-labeled β_2 protomers, as well as unlabeled β was confirmed by ESI-MS in 0.1% formic acid.

DNA Templates

DNA templates were constructed by annealing paired PAGE–purified oligonucleotides (IDT, USA; Table S1) at a molar ratio of 1:1.3 (Biotin oligo: complementary Digoxigenin or Biotin oligo) in an annealing buffer (10 mM Tris-HCl pH 8.0, 100 mM NaCl, 1 mM EDTA) at a final concentration of 5 μM. The solution was treated at 95˚C for 5 min and was then slowly cooled to room temperature over 3 h; annealed DNA substrates were stored at 4˚C. The DNA oligo modified with amino-C_6-dT was coupled with Cy3- (for smFRET) or Cy5- (for smPolarization) monofunctional NHS-esters (GE Healthcare). We mixed 5 μL of Cy3 (or Cy5) in DMSO (20 mM) and 5 μL of DNA substrates (1 mM) in 25 μL of labeling buffer (0.1 M sodium tetraborate pH 8.5) and the mixture was set aside for 6 h at room temperature. The molar ratio of the DNA substrate to the fluorophore was 1:20. Excess dye molecules were removed by ethanol precipitation; 85% of the oligonucleotides contained a single Cy3 (or Cy5), as determined using a Nanophotometer P300 spectrophotometer (IMPLEN, Germany).

Single-molecule FRET (smFRET)[S4]

DNA template (10 pM) in blocking buffer (20 mM Tris.HCl, pH 7.5, 2 mM EDTA, 0.0025% (v/v) Tween 20, 100 mg/mL BSA and 100 mM NaCl) was rapidly introduced into the sample chamber with a syringe by hand and then incubated for 30 s at room temperature without flowing. The unattached DNA molecules were removed by extensive washing with 1 ml of blocking buffer. When required, anti-digoxigenin (dig) antibody (250 nM, Roche) in the blocking buffer was linked to the 5'-dig-substituted end of the DNA by incubation in the sample chamber for 10 min.

We used a 532-nm DPSS laser (Cobolt Samba, 100 mW) to excite the donor Cy3 with 140 mW/cm^2 that was measured in front of the prism used in a prism-type total internal fluorescence (TIRF) microscope. The donor and acceptor signals were imaged in a prism-type total internal reflection fluorescence microscope (Olympus IX-71, water-type 60X objective, N.A. = 1.2) using an electron multiplying charge-coupled device (Hamamatsu ImagEM C9100-13). The image focused by the 60X objective was enlarged with a 1.6X magnifier. FRET data were analyzed using IDL (ITT VIS) and MATLAB (The MathWorks) scripts. For the time-lapse experiment, the fluorescent signals were collected with a 30 ms time resolution (excitation-on) and a 1 s time interval (excitation-off) for 90 min. To turn the excitation on and off, we used a shutter (UniBlitz), which was synchronized with the EMCCD using its internal trigger and operated by MetaMorph 7.6 (Molecular Devices). After corrections of the donor (I_D) and the acceptor (I_A) intensities for cross-talk between the donor and acceptor channels and for the background, FRET efficiencies were calculated as the ratio of I_A to $I_D + I_A$.

The experiments were carried out in a reaction buffer of 25 mM Tris.HCl, pH 7.5, 100 mM sodium chloride, 8 mM magnesium chloride, 0.1 mM EDTA and 1 mM DTT, with an oxygen scavenging system of 0.8% (w/v) D-glucose, 165 U/mL glucose oxidase and 2170 U/mL catalase (Sigma) to minimize photobleaching, and 143 mM 2-mercaptoethanol (Sigma) to suppress photoblinking of fluorophores. Although 2-mercaptoethanol can cause slow blinking of Cy5, we only observed the blinking events in 13% of β_2 loading trajectories. The frequency of the blinking in these trajectories was 1.3. Moreover, blinking events were not observed during

the short β_2 loading step. We also confirmed that the imaging buffer never induced any other FRET states except zero FRET due to photoblinking using a Cy3–Cy5 pair labeled on DNA. The resulting FRET values were 0.8 in the steady state and zero during blinking. Therefore, the fluorescence intermittency in cyanine dye had no effect on the analysis of the FRET signals.

Single–molecule fluorescence polarization (smPolarization)[S5]

The circular polarization of the excitation beam was achieved by shifting the phase of the linearly polarized laser using a quarter-wave plate (Thorlabs, USA) in front of the sample. The linearity of the beam with polarization ratio of 100:1 was improved by placing a polarizer (Thorlabs) with a polarization ratio of 10,000:1 before the quarter-wave plate. The resulting circular polarization beam shows 10% deviation between vertical and horizontal components. The fluorescence emission signals were imaged in a prism-type TIRF microscope as described above. The polarized signals from each Cy3-β_2 molecule were separated into vertically and horizontally polarized components (I_V and I_H) using the DV2 fluorescence polarization imaging system (Photometrics, DV2). We used Cy3 fluorophore as a probe for polarized states of β_2 instead of Cy5 in smFRET because Cy3 was well studied in the previous smPolarization experiments [S5].

The polarization data were analyzed using IDL (ITT VIS) and MATLAB (The MathWorks) scripts. The experiments were done in the presence of an oxygen scavenging system. Polarization was calculated as the ratio of $(I_V - I_H) / (I_V + I_H)$. The histograms of polarization were taken from the averaged single binding traces.

Table S1. Oligonucleotides of DNA Substrates for smFRET and smPolarization.

DNA duplex		Strand sequence (5' to 3')	Modification
36bp+dT_{12} DNA	36 nt	TTCTACCGACACCAG**T**GCAGTCTGATCG TGAGCTTA	5': Biotin<br>**T**: amino-C6-dT
	48 nt	TTTTTTTTTTTTTAAGCTCACGATCAGAC TGCACTGGTGTCGGTAGAA	5': Digoxigenin
75bp+dT_{10} DNA	75 nt	CCTGTTGCATCGTGCAGTTCGAGACTAG ATGTCATGCTATTCTACCGACACCAG**T**G CAGTCTGATCGTGAGCTTA	5': Biotin<br>**T**: amino-C6-dT
	85 nt	TTTTTTTTTTTAAGCTCACGATCAGACTG CACTGGTGTCGGTAGAATAGCATGACAT CTAGTCTCGAACTGCACGATGCAACAGG	5': Biotin

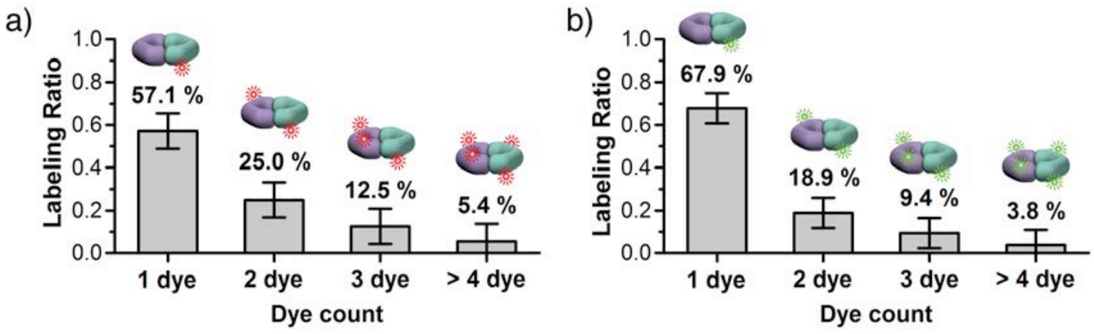

Figure S1. Labeling ratio of (a) Cy5 (N = 112) and (b) Cy3 (N = 106) on the β_2 clamp homodimer, counted using a photobleaching test in the absence of oxygen-scavenging system.

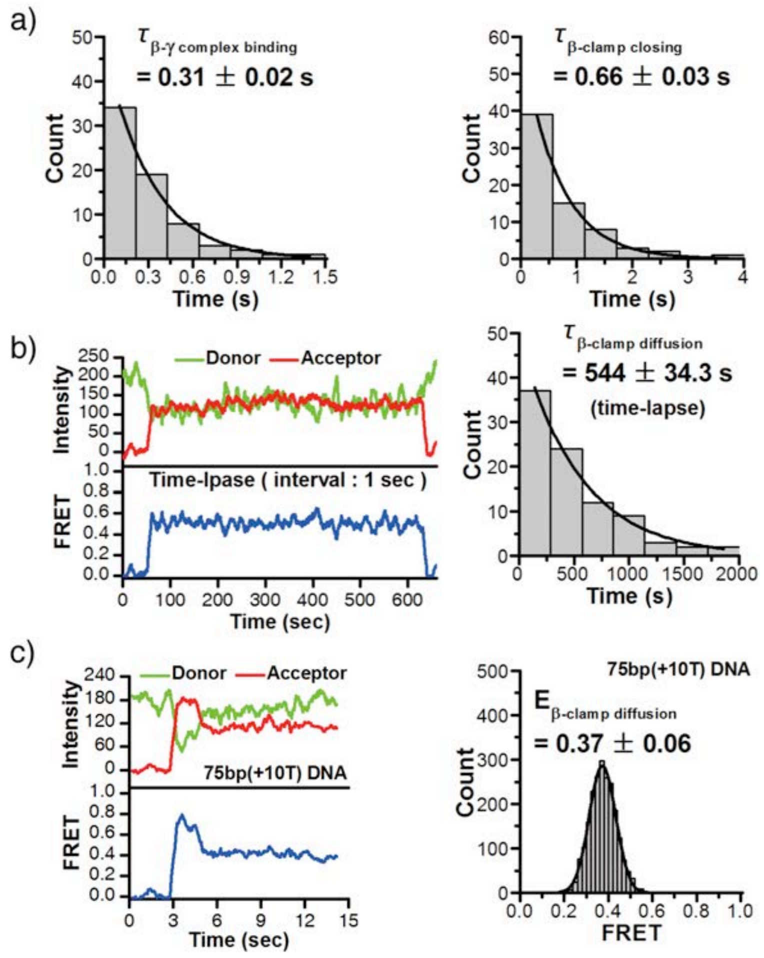

Figure S2. (a) Dwell times of two distinct FRET states in end-blocked DNA molecules with anti-dig antibody. (b) A representative time-lapse trajectory and the dwell time of β_2 clamp diffusing on end-blocked 36 bp(+dT_{12}) DNA. (c) The smFRET experiment with 75 bp(+dT_{10}) DNA showed an averaged FRET efficiency of 0.37 (± 0.06), that is lower than that on 36 bp (+dT_{12}). This result strongly supports that the β_2 clamp diffuses along DNA.

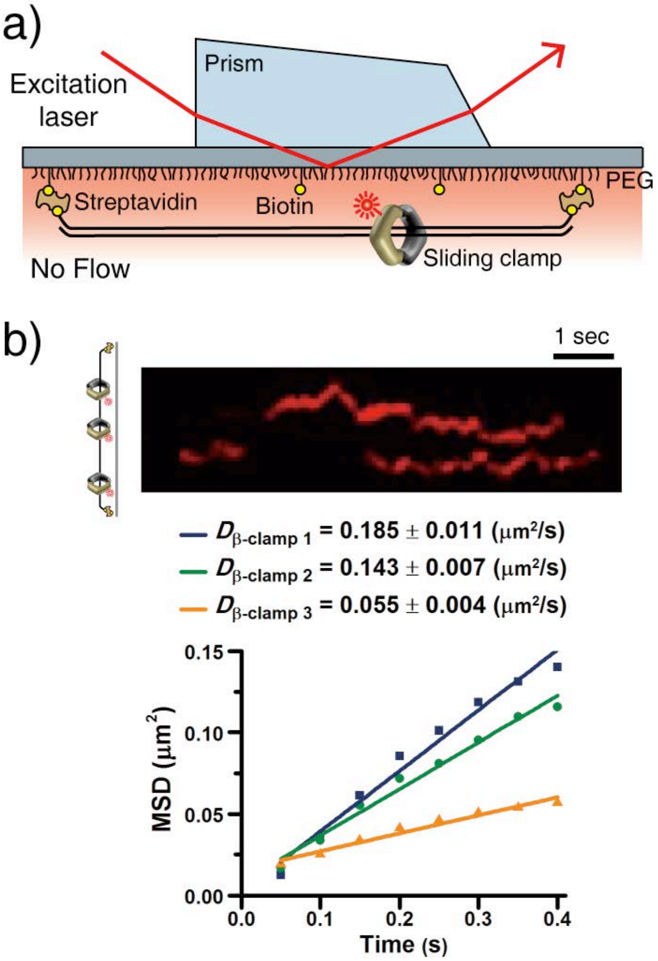

Figure S3. (a) Phage λ phage dsDNA (48.5 kb) doubly modified with biotin was stretched by a laminar flow and then immobilized. Cy5-β_2 was loaded by the clamp loader with 1 mM ATP, and in the absence of the hydrodynamic force, we imaged individual Cy5-β_2 using prism type total internal reflection fluorescence microscopy. It is likely that Cy5-β_2 is loaded at nicks in the λ DNA. (b) A kymogram for three Cy5-β_2 molecules is presented. The position of Cy5-β_2 was determined by 2D Gaussian fitting of its intensity profile. The diffusion coefficient was calculated from the slope of a mean square displacement (MSD) over the time interval. The average diffusion coefficient of Cy5-β_2 obtained from 72 molecules was measured as 0.134 $\mu m^2/s$.

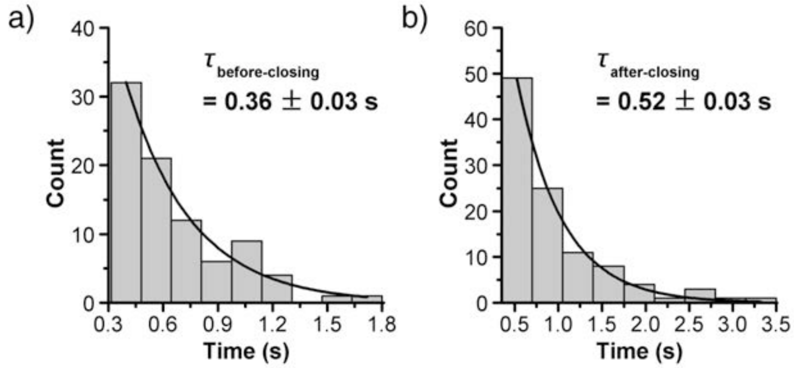

Figure S4. Dwell times of two polarized states of Cy3 on the β_2 clamp in the smPolarization experiment.

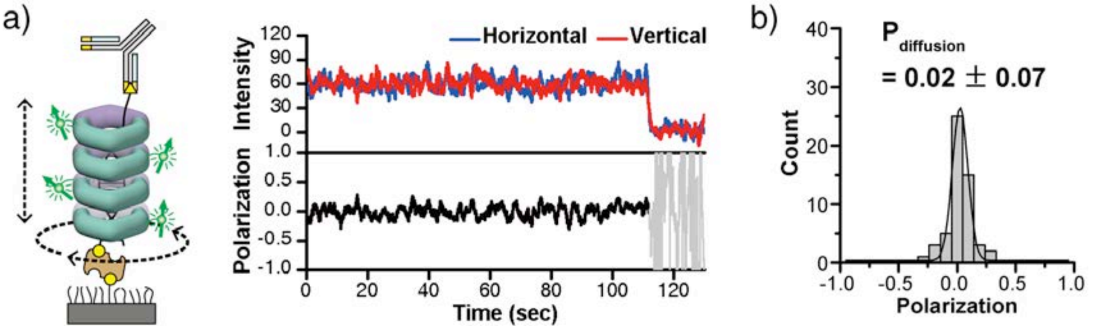

Figure S5. (a), (b) The diffusion of the β_2 clamp results in depolarized signals ($P_{diffusion}$ = 0.02 ± 0.07; mean ± s.d.). The result indicates that Cy3-β_2 rotates along DNA. Due to the limitation of the time resolution, polarized states cannot be resolved during rotation.

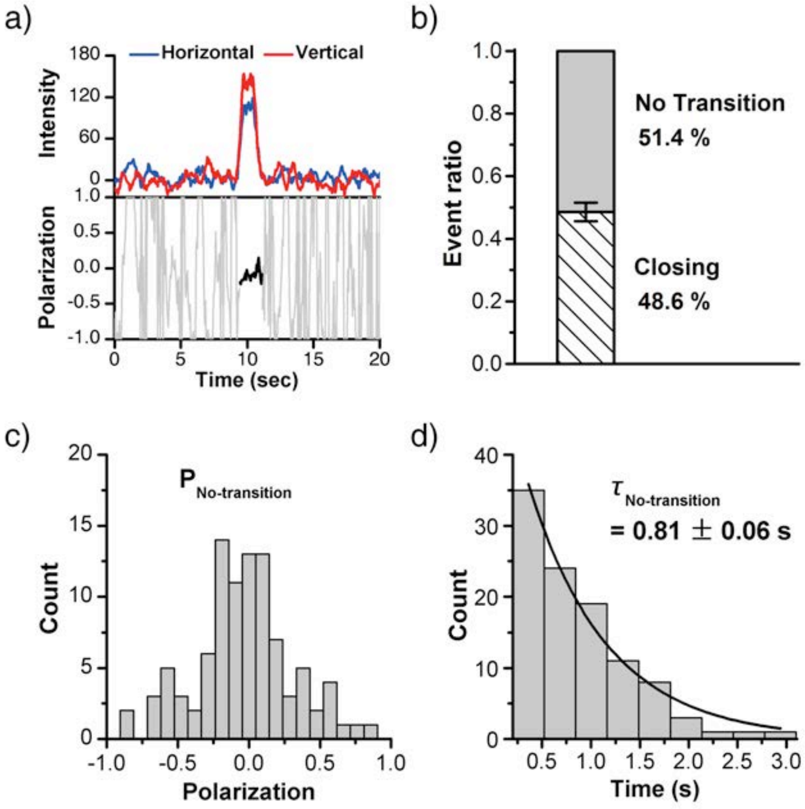

Figure S6**.** (a) A representative time trace of the fluorescence intensity in the horizontal and vertical channels and the resulting polarization, which in this case did not display the transition of polarization. (b) 48.6% of polarization traces showed the transition of two polarized states but the remaining 51.4% did not show the transition. (c) No-transition state is also polarized. (d) Dwell time of no-transition state is identical to the sum of the dwell times of the two polarized states shown in Figure S4.

REFERENCES

[S1] A. J. Oakley, P. Prosselkov, G. Wijffels, J. L. Beck, M. C. J. Wilce, N. E. Dixon, *Acta Crystallogr. D* **2003**, *59*, 1192–1199.

[S2] S. Jergic, N. P. Horan, M. M. Elshenawy, C. E. Mason, T. Urathamakul, K. Ozawa, A. Robinson, J. M. M. Goudsmits, Y. Wang, X. Pan, J. L. Beck, A. M. van Oijen, T. Huber, S. M. Hamdan, N. E. Dixon, *EMBO J.* **2013**, *32*, 1322–1333.

[S3] M. A. Griep, C. S. McHenry, *Biochemistry* **1988,** *27*, 5210–5215.

[S4] C. Jeong, W.-K. Cho, K.-M. Song, C. Cook, T.-Y. Yoon, C. Ban, R. Fishel, J.-B. Lee, *Nat. Struct. Mol. Biol.* **2011**, *18*, 379-385.

[S5] W.-K. Cho, C. Jeong, D. Kim, M. Chang, K.-M. Song, J. Hanne, C. Ban, R. Fishel, J.-B. Lee, *Structure*, **2012**, *20*, 1264-1274.
